# Humanized single-domain antibody targeting HER2 enhances function of chimeric antigen receptor T cells

**DOI:** 10.3389/fimmu.2023.1258156

**Published:** 2023-11-07

**Authors:** Rui Zheng, Yuankun Chen, Yiting Zhang, Sixin Liang, Xiaojuan Zhao, Yiyi Wang, Pengju Wang, Ruotong Meng, Angang Yang, Bo Yan

**Affiliations:** ^1^ State Key Laboratory of Holistic Integrative Management of Gastrointestinal Cancers, Department of Biochemistry and Molecular Biology, Fourth Military Medical University, Xi’an, Shaanxi, China; ^2^ College of Life Science, Shaanxi Normal University, Xi’an, Shaanxi, China; ^3^ School of Laboratory Medicine, Xinxiang Medical University, Xinxiang, Henan, China; ^4^ State Key Laboratory of Cellular Stress Biology, Innovation Center for Cell Signaling Network, School of Life Sciences, Xiamen University, Xiamen, Fujian, China; ^5^ Department of Immunology, Fourth Military Medical University, Xi’an, Shaanxi, China; ^6^ College of Life Science, Yan’an University, Yan’an, Shaanxi, China

**Keywords:** chimeric antigen receptor, antigen-binding domain, HER2, T cell engineering, adoptive immunotherapy

## Abstract

**Introduction:**

Chimeric antigen receptors (CARs) can redirect T cells against antigen-expressing tumors, and each component plays an important role in the function and anti-tumor efficacy. It has been reported that using human sequences or a low affinity of CAR single-chain variable fragments (scFvs) in the CAR binding domains is a potential way to enhance the function of CAR-T cells. However, it remains largely unknown how a lower affinity of CARs using humanized scFvs affects the function of CAR-T cells until recently.

**Methods:**

We used different humanized anti-HER2 antibodies as the extracellular domain of CARs and further constructed a series of the CAR-T cells with different affinity.

**Results:**

We have observed that moderately reducing the affinity of CARs (light chain variable domain (V_L_)-based CAR-T) could maintain the anti-tumor efficacy, and improved the safety of CAR therapy both *in vitro* and *in vivo* compared with high-affinity CAR-T cells. Moreover, T cells expressing the V_L_ domain only antibody exhibited long-lasting tumor elimination capability after multiple challenges *in vitro*, longer persistence and lower cytokine levels *in vivo*.

**Discussion:**

Our findings provide an alternative option for CAR-T optimization with the potential to widen the use of CAR T cells.

## 1 Introduction

T lymphocytes are critical cells of the immune system for anti-tumor immunity. Chimeric antigen receptor (CAR)-engineered T cells have been developed as the main cellular therapy to redirect antigen recognition, mediate T-cell activation, and induce potent anti-tumor activity (1, 2). In recent years, adoptive T-cell immunotherapy has achieved promising therapeutic efficacy in treating hematological cancers (3, 4). Previous studies have shown that CAR-T cells exert potent anti-tumor activity across a wide range of affinities and that the affinities of CAR-T cells used in clinical treatment likely exceed the required affinity threshold (5, 6). Many current strategies for generating CARs consist of selecting single-chain variable fragments (scFvs) with high affinity, as previous studies have shown that the activation threshold is inversely proportional to the affinity of the scFv (5, 7). However, the high affinity of CARs may result in severe adverse events associated with the treatment of cancer patients with CAR-T cells. For example, the high affinity of CAR-T cells targeting ErbB2 [human epidermal growth factor receptor 2 (HER2)] or ROR1 led to serious toxicity due to the recognition of target antigens on normal tissues (8, 9). Thus, it is crucial to select CARs with appropriate affinity for safe and efficient therapy.

CARs are synthetic immune receptors that are designed with an extracellular antigen-recognition domain, a hinge and transmembrane domain, and an intracellular activation domain that consists of one to two co-stimulatory domains and CD3ζ (10, 11). Each component of CARs contributes to the function and anti-tumor efficacy of CAR-T cells (12, 13). HER2 is overexpressed in various malignancies and a small subset of tissues, and this restricted expression pattern makes HER2 an efficient, highly selected target antigen for immunotherapies in multiple malignant tumors (6, 14, 15).

scFvs, commonly known as antigen-binding domains, confer non-MHC-restricted recognition of tumor cells, as well as initiate and determine the strength of T-cell activation (16, 17). Generally, high-affinity scFvs endow T cells with stronger anti-tumor activity than low-affinity scFvs in CAR-T cells. Nevertheless, the affinity of CARs beyond a certain level may be harmful and may not further enhance the function of CAR-T cells (5). For instance, it may increase the risk of on-target/off-tumor toxicity, which eliminates more normal cells with lower antigen density and can sometimes be fatal to patients (7, 17, 18). One practical way to avert the “on-target/off-tumor” reactivity of CAR-T cells is to decrease the affinity of scFvs in CARs without reducing their killing capability (19–21). However, an excessive decrease in CAR affinity may result in a higher target expression threshold for T-cell activation and hinder the efficacy of the anti-tumor function of CAR-T cells (5, 7). Therefore, it is crucial to identify the conditions for CAR affinity to achieve the best discriminative potential in combination with an optimal anti-tumor effect.

Nowadays, the scFvs of CARs used frequently in clinical trials have a murine origin that may be limited by the induction of transgene immunogenicity, resulting in poor persistence and function of CAR-T cells *in vivo* (22–24). One way to potentially reduce the immunogenicity of CAR binding domains is to use a human sequence instead of a murine sequence (25–27). We previously humanized the HER2-targeting scFvs using a human antibody consensus sequence to replace the murine origin scFvs of HER2 (e23sFv), and the results showed that humanized scFvs (P1h2 and P2h2) improved the HER2-targeting and tumor-killing capacities compared to the murine origin antibody (28). However, the ability of these proteins to respond to HER2 antigen loading into CAR-T cells has not been systemically investigated.

The constructs of scFvs consist of a light variable chain and a heavy variable chain (V_L_ and V_H_, respectively) of monoclonal antibodies, and they are fused via a short flexible linker (17, 29). Recently, research in CAR-T therapy has revealed that T cells redirected based on an immunoglobulin heavy-chain variable region domain have been proven to maintain a strong and specific antigen-binding ability (30–32). Accumulating evidence from CAR-T cells with changes in antigen-binding domains has demonstrated that only single-domain antibodies (sdAbs), comprising the variable domain of V_L_ or V_H_, can be used to generate functional CAR molecules and redirect T cells to elicit anti-tumor responses (21, 33, 34). Moreover, the smaller size of a heavy-chain-only antigen-recognition domain exhibited advantages in antigen rearrangement and expression in CAR-T cells (27, 30). However, the ability of humanized anti-HER2 proteins in CAR therapy has not been systematically investigated.

Here, we performed a new affinity-tuning approach for generating low-affinity scFv variants derived from the selection of a HER2-targeting humanized murine scFv. In this study, we established a series of anti-HER2 scFv antibodies derived from our previous mutant pool of humanized anti-HER2 single-chain variable fragments (28). Then, we used these antibodies to construct a panel of CAR-T cells with distinct affinities only for the extracellular binding domains. Briefly, we incorporated these sdAb and scFvs genes with CD8 hinge/TM, CD28, 41BB, and CD3ζ intracellular signaling domains. Then, we evaluated the anti-tumor functionality, phenotype, expansion, and persistence of the generated CAR-T cells both *in vitro* and *in vivo*. We found that low-affinity CAR-T cells showed increased expansion, maintained anti-tumor activity *in vitro* and *in vivo*, and potentially augmented CAR-T cell persistence *in vivo*.

## 2 Materials and methods

### 2.1 Generation of sdAbs and scFv antibodies

A panel of humanized anti-HER2 antibodies (sdAb) was produced according to the description from our previous study (28), comprising variable domains of the heavy chain (V_H_) or light chain (V_L_). P1h2 and P2h2, which have the same V_L_ domain and V_H_ domains, were made in two formats: V_H1_ from P1h2 and V_H2_ from P2h2. Briefly, 293F cells were cultured in CDM4HEK293™ (Cat# SH30858.02, Cytiva, Marlborough, MA, USA) and transfected with plasmid using FectoPRO (Cat# 101000019, Polyplus, Illkirch, France) to produce proteins when the cell density reached 1 × 10^6^/mL. The cells were incubated at 37°C in 8% CO_2_ for 6 days, and the supernatant was collected by centrifugation. Then, the supernatant was purified by Ni-NTA binding. Bound proteins were washed with 30 mM imidazole and eluted with 100 mM imidazole, and the eluates were dialyzed against phosphate-buffered saline (PBS). Amicon® Ultra-15 (Cat# UFC9010, Millipore, Billerica, MA, USA) was used to condense the protein, and Pierce™ BCA kit (Cat# 23227, Thermo Fisher Scientific, Waltham, MA, USA) was used to measure the concentration of protein. Then, the different proteins were diluted to 1 mg/mL using PBS.

### 2.2 Surface plasmon resonance

Surface plasmon resonance (SPR) was used to determine the kinetics of binding between the recombinant human HER2 (rhHER2) *in vitro*. Binding analyses were performed using the Biacore T200 at a temperature of 25°C. Briefly, rhHER2 was diluted to 5 mg/mL and then immobilized on a CM5 sensor chip (GE Healthcare Life Sciences, Pittsburgh, PA, USA). The diluted proteins (V_L_, V_H1_, V_H2_, P1h2, P2h2, and e23) were subsequently injected into the chip. Each sample was run through for a duration of 100 s at a flow rate of 30 µL/min and dissociated for 200 s. The antigen surface was regenerated at a flow rate of 10 µL/min by 30-s injection of 10 mM glycine at pH 2.5.

### 2.3 Cell lines and culture conditions

293T and HeLa cells were obtained from the American Type Cell Culture Collection (ATCC), and the PC9 cell line was purchased from Pricell Life Science & Technology Co., Ltd. (Wuhan, China). 293T cells were used to produce the lentivirus by transfecting the lentiviral vectors into cells. The tumor cell lines HeLa and PC9, which expressed stable levels of human HER2 antigens, were used as target cells. For the luciferase-based cytolysis assay, HeLa-luciferase and PC9-luciferase cell lines were generated by transducing the lentivirus containing the firefly luciferase genes into the cell lines (**Figures S2C, D**). Dulbecco’s modified Eagle medium (DMEM) (Gibco, Grand Island, NY, USA) and Roswell Park Memorial Institute (RPMI) 1640 (Gibco) supplemented with 10% fetal bovine serum (FBS) (Lonza, Hopkinton, MA, USA) were used to culture the HeLa and PC9 cells, respectively. Trypsin from porcine, 1:250 (Cat# DY40116, DIYIbio), was used for cell dissociation at a concentration of 0.25% with 0.1% ethylenediaminetetraacetic acid (EDTA). The 293T cells were digested for 1 min at 37°C, while PC9 and HeLa cells were digested for 2 min at 37°C. The 293T cells were maintained in DMEM (Gibco) supplemented with 10% FBS and 1% l-glutamine. All cells were incubated at 37°C in 5% CO_2_.

### 2.4 Primary human T-cell isolation and CAR-T cell generation

The healthy volunteers who provided the blood were from our own group (samples were collected between June and August 2022), including one man and three women, aged 25–32 years, with an average age of 27 years. All donors provided informed written consent, and the use of human peripheral blood was approved (Approval ID: KY20214016-1) by the Ethics Committee of the Fourth Military Medical University (Xi’an, China). Peripheral blood mononuclear cells (PBMCs) were isolated from healthy donors using Lymphoprep density separation (Ficoll, Kewei), and the primary human T cells were isolated from PBMCs using the MojoSort™ Human CD3 T-Cell Isolation Kit according to the manufacturer’s instructions (Cat# 480131, BioLegend, San Diego, CA, USA). Then, the primary T cells were stimulated with Activation/Expansion CD3/CD28 Beads (Cat# MBS-C001, ACROBiosystems, Newark, DE, USA). T cells were cultured in X-VIVO15 medium supplemented with 10% FBS, 1% penicillin and streptomycin sulfate, and 100 IU/mL recombinant human IL-2 (Cat# 200-02-100, PeproTech, Cranbury, NJ, USA). After 24 hours of activation, T cells were transduced with lentiviral supernatants containing different CAR genes.

The lentiviral supernatant was produced by the 293T cell line using the pMD2.G and psPAX2 packaging system (35). Then, the lentiviral supernatant was condensed using 3 M NaCl and PEG 6000. Briefly, approximately 20 mL of CAR lentiviral supernatant was collected; then, 0.137N 3 M NaCl and 0.233N PEG 6000 were added to the tube and kept at 4°C for 1.5 hours. Then, the solution was centrifuged at 7,000 *g* for 30 min, the supernatant was discarded, and the mixture was centrifuged again for 5 min. Ultimately, the lentivirus sediments were resuspended in 200 μL of RPMI 1640 (Gibco).

### 2.5 *In vitro* T-cell cytotoxicity assessment

In the bioluminescence imaging (BLI)-based cytotoxicity assay, target tumor cells were seeded overnight in a black 96-well plate with a clear bottom at a concentration of 1 × 10^4^ cells/well. Then, CAR-T cells were added to the plate at different effector-to-target (E:T) ratios from 8:1 to 1:4. Cocultures were analyzed 14 h after coculture to measure residual tumor cells, which were monitored by bioluminescence using IVIS Lumina II (Caliper, Waltham, MA, USA). Moreover, CAR-T cells and target tumor cells were cultured at an E:T ratio of 2:1 for 30 h and monitored by bioluminescence. For the re-stimulation experiment, the cells were exposed to the antigen continuously (CAE) once every 4 days for a total of 20 days. Specific lysis was calculated using the following formula:


$$\text{\% Lysis=}\frac{\text{spontaneous lysis-experimental lysis}}{\text{spontaneous lysis}}\times 100\%$$

For observation, the tumor cells were stained with carboxyfluorescein succinimidyl ester (CFSE) (Invitrogen, Carlsbad, CA, USA) according to the manufacturer’s protocol. Briefly, tumor cells were digested with pancreatin and washed with PBS three times. Then, the cells were resuspended in PBS at a concentration of 5 × 10^6^/mL, and CFSE was added (final concentration of 2 μM). They were incubated for 10 min before adding 1640 with 10% FBS to stop staining. The cells were then washed with PBS three times, counted, and seeded overnight in a 96-well plate at a concentration of 5 × 10^4^ cells/well. CAR-T cells were counted and added to the plate at an E:T ratio of 2:1. Finally, the target tumor cells were observed using fluorescence microscopy (Leica, Wetzlar, Germany) at 16 h and 30 h.

### 2.6 Proliferation assay

A total of 5 × 10^5^ CAR-T cells were counted and plated into a 12-well plate along with HER2^+^ tumor cells at an E:T ratio of 5:1. Subsequently, CAR-T cells were counted every 3 days and stimulated every 6 days using target tumor cells. Additionally, on days 6 and 12, CAR-T cells were harvested and analyzed using Ki67 staining (APC anti-human Ki-67 Antibody, BioLegend) by flow cytometry.

### 2.7 Flow cytometry

Flow cytometry was performed using CytoFLEX (Beckman Coulter, Brea, CA, USA). Data analysis was performed using FlowJo V10. CAR efficiency was measured using a biotinylated rhHER2 protein and stained with APC-streptavidin (1:20, Cat# 405207, BioLegend). In addition, the antibodies used in flow cytometry were as follows: mAbs for Alexa Fluor® anti-human CD340 (erB2/HER-2) (1:20, Cat# 324410, BioLegend), human CD3 (1:20, Cat# 300305, BioLegend), CD25 (1:20, Cat# 985802, BioLegend), CD69 (1:20, Cat# 310910, BioLegend), PD-1 (1:20, CD279, Cat# 379210, BioLegend), LAG3 (1:20, CD223, Cat# 369212, BioLegend), TIM3 (1:20, CD366, Cat# 345026, BioLegend), CD45RA (1:20, Cat# 304108, BioLegend), CD62L (1:20, Cat# 304814, BioLegend), CD107a (LAMP-1, 1:20, Cat# 328607, BioLegend), and Perforin (1:20, Cat# 308106, BioLegend). Moreover, Annexin V (1:20, Cat# 640951, BioLegend) and its binding buffer (Cat# 422201, BioLegend), 7-AAD (1:20, Cat# 420404, BioLegend), Granzyme B (1:20, Cat# 372206, BioLegend) were used. A total of 1 × 10^6^ cells per group were collected, washed by PBS three times, and then stained and underwent 30-min incubation at 4°C in the dark. For apoptosis staining, cells were collected, washed by PBS three times, and then stained using Annexin V with its binding buffer (5 μL Annexin V in 100 μL binding buffer) for 15-min incubation at room temperature. For intracellular staining, cells were permeabilized and fixed by Cyto-Fast™ Fix/Perm Buffer Set according to the manufacturer’s instructions (Cat# 426803, BioLegend).

### 2.8 Cytokine analysis

*In vitro*, a total of 2 × 10^4^ CAR-T cells were incubated with 2 × 10^4^ HER2^+^ HeLa in 96-well plates without exogenous cytokines. The supernatant was collected after 12 hours of coculture, and cytokines (TNF-a, IL-2, INF-γ, GM-CSF, and IL-6) were measured using LEGENDplex™ HU Cytokine Release Syndrome Panel (13-plex) w/VbP (Cat# 740930, BioLegend) according to the manufacturer’s protocol.

### 2.9 *In vivo* tumor killing and mouse models

The animal experiments were conducted in compliance with the approval of the Fourth Military Medical University Ethics Committee on Animal Care (Approval ID: 20210565). In the present study, the humane endpoints were as follows: if the 1) tumor volume was greater than 1 cm^3^ or 2) the mice lost 20% of their body weight over the course of treatment, the mice were euthanized. NCG (NOD-Prkdc^em26Cd52^Il2rg^em26Cd22^/Nju) mice (4–6 weeks old) were purchased from GemPharmatech Co., Ltd. (Nanjing, China) (36) and used to generate human xenograft tumor models. Mice were injected subcutaneously (s.c.) with 5 × 10^5^ HER2^+^Fluc^+^ HeLa cells per mouse. Before the CAR-T cells were injected, the mice were randomized on the basis of tumor radiance. Subsequently, 7.5 × 10^5^ CAR-T cells were injected intravenously through the tail vein (i.v.) per mouse after 5 days of tumor injection. Bioluminescence images were acquired and analyzed using the IVIS Imaging System. To track CAR-T cells, 100 μL of RediJect Coelenterazine was injected intravenously (i.v.) per mouse (P/N 760564, PerkiEime). To track tumor cells, 100 μL of d-luciferin was injected intraperitoneally (i.p.) per mouse. Photon emission from tumor cells was expressed as photons per second per cm^2^ per steradian. Before imaging, mice were anesthetized by inhalation of 2% isoflurane medical oxygen (Zoetis) and maintained under continuous inhalational anesthesia during imaging.

For cytokine analysis, 14 days after CAR-T cell injection, the peripheral blood of mice was collected from the tail vein (100 μL per mouse). Then, the blood was centrifuged at 3,000 rpm for 5 min; this step was repeated three times to obtain the serum of mice. Then, the human and murine cytokines were measured using LEGENDplex™ HU Cytokine Release Syndrome Panel (13-plex) w/VbP (Cat# 740930, BioLegend) and LEGENDplex™ MU Cytokine Release Syndrome Panel (13-plex) w/VbP (Cat# 741024, BioLegend) according to the manufacturer’s protocol, respectively.

### 2.10 Hematoxylin and eosin and immunohistochemistry analysis

On day 12 after CAR-T cell injection, the mice were sacrificed by intraperitoneal injection of 1% pentobarbital sodium (200 mg/kg). The tissues (including the spleen, lung, liver, kidney, and heart) were carefully excised from the body, dehydrated, and embedded in paraffin wax for hematoxylin and eosin (H&E) staining and immunohistochemistry. For immunohistochemistry, the tumor tissues were collected, fixed, processed, and stained according to standard procedures. Briefly, the tissues were fixed in 4% paraformaldehyde at room temperature for 24 h, embedded in paraffin wax, and sectioned to a thickness of 4 μm. Xylene and graded concentrations of ethanol were used to dewax and hydrate the tissue sections. Then, hydrogen peroxide (3%) and sodium citrate buffer (0.01 M, pH = 6) (Cat# G1202-250ML, Servicebio, Wuhan, China) were used to block endogenous peroxidase and for antigen retrieval, respectively. After blocking in 1% bovine serum albumin for 30 min at room temperature, the slides were incubated with primary antibodies at 4°C overnight, including CD3 (1:5,000, Cat# 60181-1-Ig, ProteinTech, Chicago, IL, USA). The next day, the slides were incubated with horseradish peroxidase (HRP)-conjugated secondary antibodies (1:200, Cat# G1214-100UL, Servicebio). Finally, sections were visualized, and images were acquired using a fluorescence continuous scanning digital section microscope (OLYMPUS VS200).

### 2.11 Statistical analysis

IBM SPSS 23 (IBM, Chicago, USA) was used for statistical analysis. The data are expressed as the means ± SD for both *in vitro* and *in vivo* experiments and visualized using GraphPad Prism version 8.0. One-way ANOVA followed by least significant difference (LSD) multiple comparison tests (n< 4) or Dunnett’s T3 multiple comparison tests (n ≥ 4) were used to calculate the p-values. The significance of the findings is defined as follows: ns, not significant; ^*^p ≤ 0.05; ^**^p ≤ 0.01; ^***^p ≤ 0.001. The survival curves were analyzed using the log-rank (Mantel–Cox) test.

## 3 Results

### 3.1 Analysis of binding affinities for anti-HER2 sdAbs or scFvs

**Figure 1:**
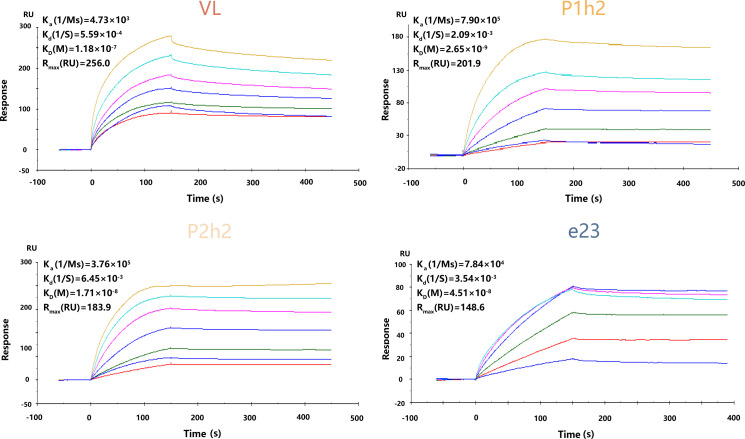
The kinetics of recombinant proteins *in vitro*. SPR kinetic rates and apparent affinity binding constants of HER2 scFv in VL-CAR, P1h2-CAR, P2h2-CAR, and e23-CAR groups. The binding affinities (K_D_) constants that resulted from the fit are shown. All experiments were carried out at a constant flow rate of 10 μL/min. SPR, surface plasmon resonance.

The anti-HER2 sdAbs or scFvs were purified using Ni-NTA binding, and Coomassie bright blue staining showed that all proteins were purified successfully from the protein stock solution (**Figure S1A**). In addition, it was observed that V_L_, P1h2, and P2h2 were highly expressed compared with V_H1_ and V_H2_ (**Figure S1A**). The results from the SPR assay showed that V_H1_ and V_H2_ could not specifically bind to the recombinant HER2 antigens (**Figure S1B**). Hence, the humanized anti-HER2 sdAbs or scFvs of V_L_, P1h2, and P2h2 were chosen for the further construction of CAR molecules. The affinities of these antibodies were P1h2>P2h2>e23>V_L_, with affinities ranging from 2.65 nM to 118 nM (**Figure 1**).

### 3.2 VL-CAR-T exhibited a robust ability in proliferation *in vitro*

**Figure 2:**
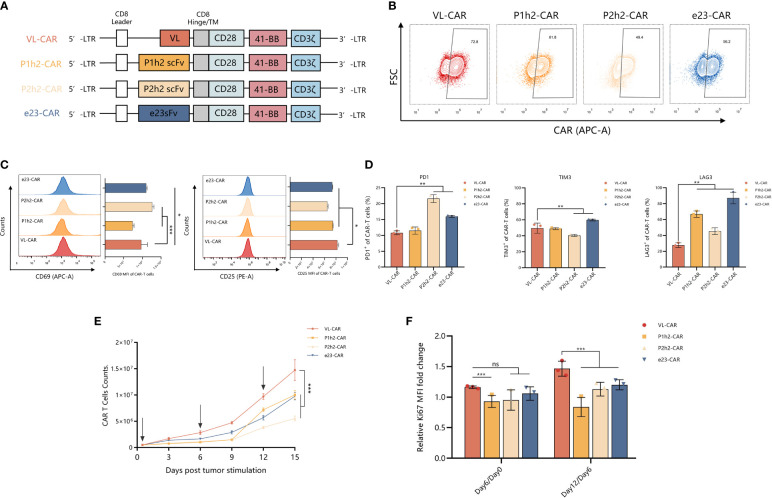
VL-CAR-T exhibited a robust ability in proliferation *in vitro*. **(A)** Schematic diagram of VL, P1h2, P2h2, and e23 CAR constructs. Characterization of **(B)** CAR expression, **(C)** markers of activation, and **(D)** markers of inhibition in CAR-T cells using flow cytometry. **(E)** Cell counts of CAR-T cells; the arrows represent HER2-positive tumor cell stimulation. **(F)** Rates of T-cell growth *in vitro*. Bars indicate the mean ± SD. Asterisk indicates a statistically significant difference compared to the VL-based CAR-T group, ns, no significance, *p ≤ 0.05, **p ≤ 0.01, ***p ≤ 0.001.

We designed and constructed four sdAb- or scFv-based CAR-T cells using different humanized HER2 binding proteins and linked them to the CD8α stalk, the two most popular co-stimulatory domains (CD28 and 4-1BB), and CD3ζ intracellular domain (**Figure 2A**). We transduced activated T cells isolated from the peripheral blood of healthy donors with CAR genes for CAR-T preparation, and the results showed that >55% of the T cells were transgene-positive, as evidenced by the efficiency of CAR expression on the surface of T cells (**Figure 2B**). Furthermore, we measured the state of activity of the variant CAR-T cell products, including markers of activation and inhibition in T cells, using flow cytometry *in vitro*. We observed a significantly increased expression of activated markers, suggesting that there is a strong activation of VL-CAR-T cells compared with other CAR-T groups (**Figure 2C**). In addition, we found that the percentage of inhibitory markers (including PD1, TIM3, and LAG3) was significantly decreased in the V_L_-based CAR-T cells compared with the others (**Figures 2D**, **S2A**). Additionally, we found that there was a similar expansion trend for V_L_-based and other CAR-T cells but a significant increase in cell counts when stimulated by the positive antigen (**Figure 2E**). Similarly, we observed that the expression of Ki67, a marker of proliferation, was significantly increased in the V_L_-based CAR-T cells compared with other CAR-T cells (**Figure 2F**).

### 3.3 VL-CAR T can enhance the persistence and anti-tumor activity *in vitro*

**Figure 3:**
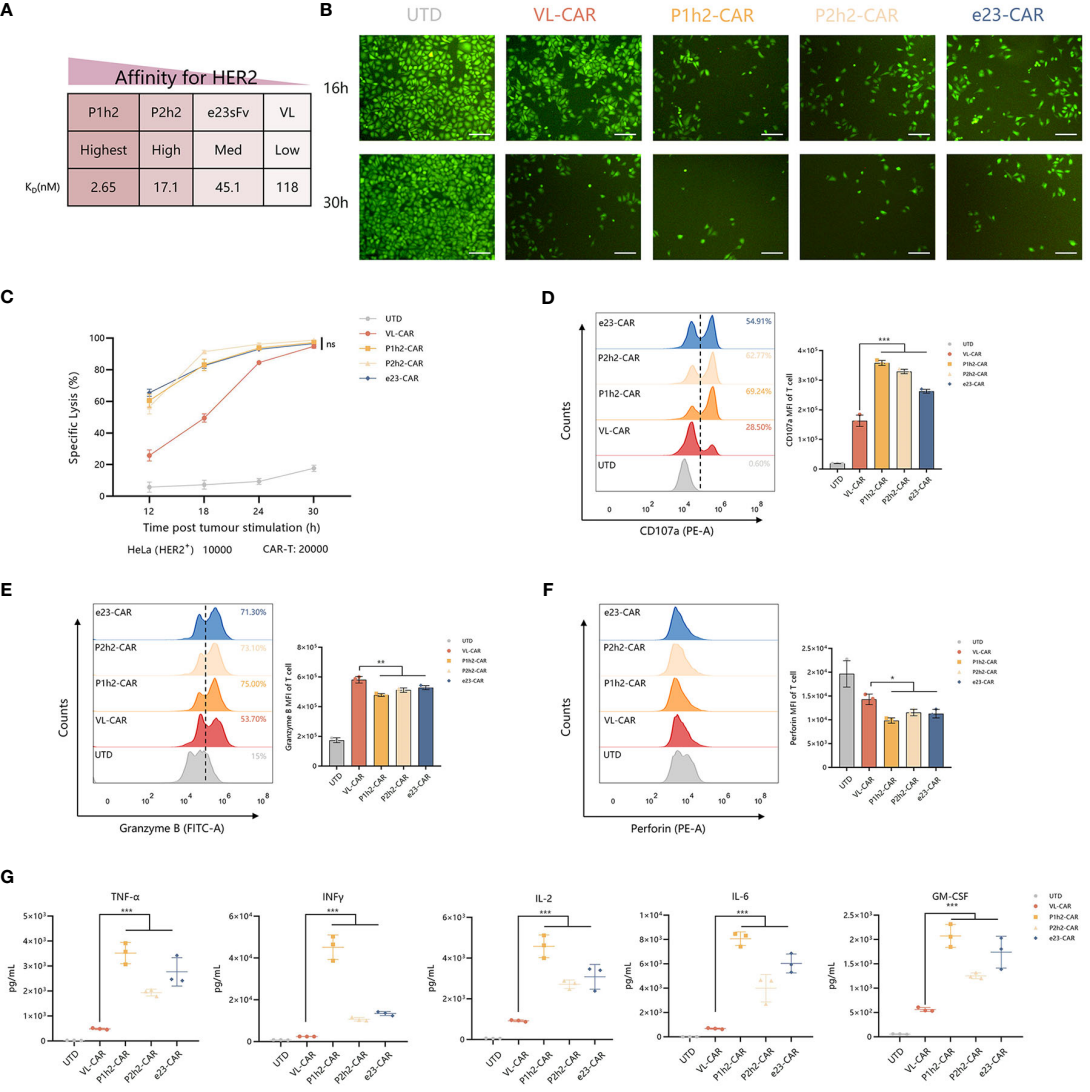
VL-CAR T can enhance the persistence and anti-tumor activity. **(A)** Affinity characteristics are shown for humanized anti-HER2 sdAbs and scFvs. CAR-T cells were sorted and then cocultured with tumor cells. **(B)** Representative images of CFSE in HER2^+^ tumor cells cocultured with VL or scFv-based CAR-T at the E:T ratio of 2:1 for 16 h and 30h. Scale bar = 100 μm. **(C)** The series CAR-T cells were incubated with firefly luciferase-transduced HER2^+^ HeLa cells at the E:T ratio of 2:1. Points represent mean cytotoxicity of replicate wells ± SD. Representative flow cytometry plots illustrating the expression of CD107a **(D)**, Granzyme B **(E)**, and Perforin **(F)** in T cells incubated with HER2^+^ HeLa cells for 6h. **(H, G)** Cytokine secretion for coculture supernatant (E:T ratio of 2:1) at 12h. Asterisk indicates a statistically significant difference compared to the VL-based CAR-T group, ns, no significance, *p ≤ 0.05, **p ≤ 0.01, ***p ≤ 0.001. sdAbs, single-domain antibodies; scFvs, single-chain variable fragments; CFSE, carboxyfluorescein succinimidyl ester.

To evaluate the precise recognition of VL-CAR-T cells, we sorted the CAR-T cells using HER2-coated beads and ensured that the CAR-positive cells reached more than 90%. Then, we measured the killing ability of different CAR-T cells by coculturing them with HER2^+^/HER2^−^ cells (**Figure 3A**). The results of CFSE showed that CAR-T cells of all constructs could precisely recognize HER2-positive cells and had no effect on HER2-negative cells (**Figures 3B**, **S2D**). Although there was a weak killing efficacy during the short time of coculture in VL-CAR-T cells at all listed E:T ratios (**Figure S2E**), there were no significant differences in specific lysis between VL-CAR T and other CAR-T cells as time progressed (**Figures 3B, C**). We observed the same pattern in PC9 cells artificially engineered to express HER2 as target cells (**Figure S2F**). In the CD107a degranulation assays, we detected lower degranulation and release of CD107a in VL-CAR-T cells compared with scFv-based CAR-T cells (**Figure 3D**). In addition, the levels of intracellular Granzyme B and Perforin were significantly increased in VL-CAR-T cells compared with scFv-based CAR-T cells after coculturing with HER2^+^ HeLa cells (**Figures 3E, F**). In a similar fashion, V_L_-based CAR-T cells displayed significantly lower secretion of effector cytokines TNF-α, IL-2, INF-γ, GM-CSF, and IL-6 in the supernatant compared with scFv-based CAR-T cells (**Figure 3G**).

### 3.4 VL-CAR T exhibited higher cytotoxicity *in vitro* under multiple rounds of antigen stimulation

**Figure 4:**
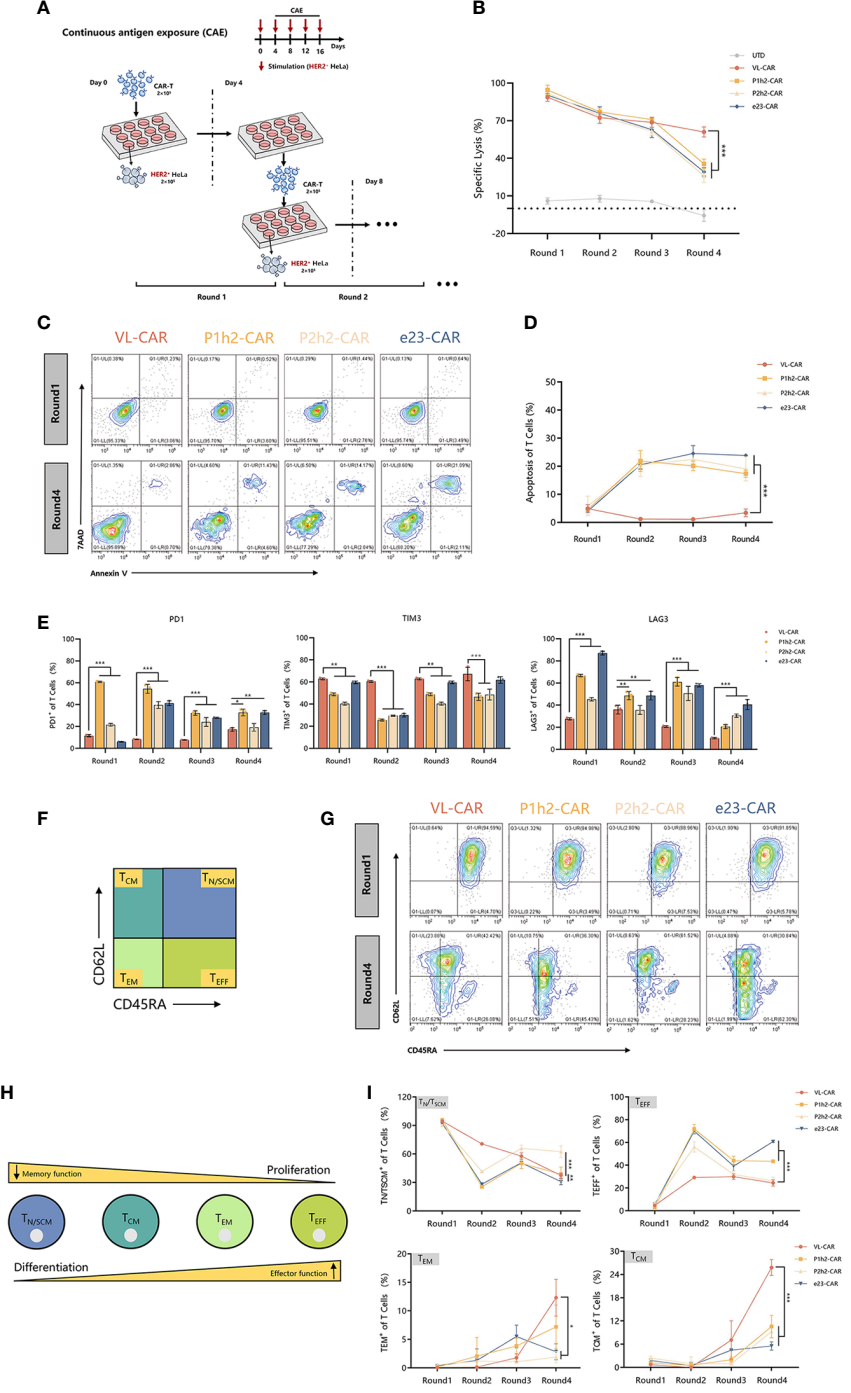
Fitness of CAR-T cell to repeated antigen stimulation assays *in vitro*. CAR-T cells were sorted and then cocultured with tumor cells. **(A)** Scheme of re-stimulation of CAR-T cells with HER2^+^ target cells. **(B)** *In vitro* cytotoxicity of CAR-T cells in CAE experiment using luciferase assay. **(C, D)** Apoptotic characterization of CAR-T cells by Annexin V/7-AAD binding assessment during CAE. **(E)** Representative flow cytometry plots illustrating the expression of exhausted markers including PD1, LAG3, and TIM3 during CAE experiment. **(F)** Different subsets of T cells. **(G–I)** Phenotype of CAR-T cells stimulated repeatedly in CAE experiment using flow cytometry. Asterisk indicates a statistically significant difference compared to the VL-based CAR-T group, ns, no significance, *p ≤ 0.05, **p ≤ 0.01, ***p ≤ 0.001.

We stimulated different CAR-T cells with target cells at a 2:1 E:T ratio for four rounds because enduring multiple antigen challenges is one of the evaluation measures in the killing functions of CAR-T cells (**Figure 4A**). The experiment of re-stimulating CAR-T cells with HER2^+^ target cells occurred every 4 days for a total of 16 days. We observed that there were no significant differences in killing ability between VL-CAR-T cells and other CAR-T groups in the first three rounds of stimulation, while there was a significant increase in tumor lysis in the VL-CAR-T cells to HER2^+^ target cells compared to scFv-based CAR-T cells (**Figure 4B**). Further, to explore whether the decreased affinity of CARs could rescue T-cell exhaustion, we detected the percentage of apoptosis and exhaustion markers of CAR-T cells using flow cytometry. The results showed that a lower percentage of apoptosis was observed in the VL-CAR-T cell group during the all-CAE rounds of the experiment compared with other scFv CAR-T cells (**Figures 4C, D**). Similarly, VL-CAR-T cells showed reduced expression of T-cell exhaustion-related markers during the CAE experiment compared with scFv-CAR-T cells (**Figure 4E**).

Previous studies have shown that the subtypes of T cells are critical for long-term persistence and important for *in vivo* efficacy. Accordingly, we determined the phenotypic markers of CAR-T cells during the multiple antigen challenges using flow cytometry (**Figures 4F, H**). After stimulation, there was a significantly increased percentage of memory T cells [including T central memory cells (T_CM_, CD45^−^RACD62L^+^) and T effector memory cells (T_EM_, CD45RA^−^CD62L^−^)] while a significantly decreased percentage of T effector cells (T_EFF_, CD45RA^+^CD62L^+^) and stem cell memory T cells (T_N_/T_SCM_, CD45RA^+^CD62L^−^) in VL-CAR-T cells compared with the scFv-based CAR-T cells *in vitro* (**Figures 4G, I**).

### 3.5 VL-CAR-T cells show long-term persistence and anti-tumor activity *in vivo*

**Figure 5:**
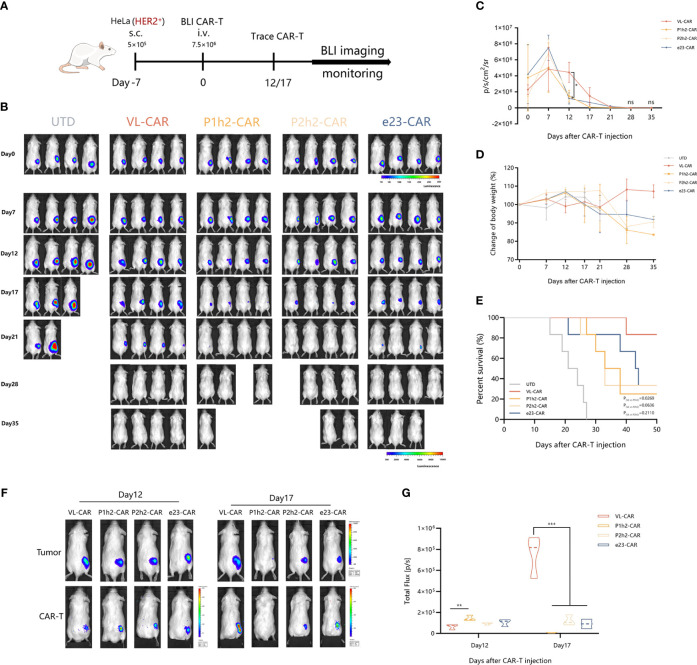
VL-CAR-T cells show long-term persistence and anti-tumor activity *in vivo*. **(A)** Schematic of the subcutaneous injection tumor model using HER2^+^ HeLa cells in NCG mice and treatment with four different nanoluc-CAR-T cells. **(B)** Bioluminescent images of each mouse per condition at specified days after UTD/CAR-T cell injection. Averaged **(C)** tumor growth curves and **(D)** body weight for all mice in the different CAR-T cell treatments. **(E)** Kaplan–Meier survival curve of mice treated with UTD/CAR-T cell. **(F)** Images of CAR-T cells at selected time points and **(G)** BLI kinetics of CAR-T cells post T-cell injections (n = 3 per group). Asterisk indicates a statistically significant difference compared to the VL-based CAR-T group, ns, no significance, *p ≤ 0.05, **p ≤ 0.01, ***p ≤ 0.001. BLI, bioluminescence imaging.

NCG mice engrafted with HER2^+^ HeLa (5 × 10^5^ cells/mouse) labeled with firefly luciferase and were treated with different CAR-T cells (7.5 × 10^5^ cells/mouse) after 7 days of tumor cell injection (**Figure 5A**). As the tumor bioluminescence results showed, treatment with V_L_-based CAR-T cells did not result in a significant delay in tumor progression in the first 2 weeks. However, the overall survival of mice treated with VL-CAR-T cells was longer, and tumor growth was eventually controlled compared to other CAR-T treatment groups. Moreover, there was no statistical difference in tumor burdens between VL and other CAR-T cells in mice after 3 weeks of treatment (**Figures 5B, C**, **S3A**). Additionally, an increase in body weight was observed in mice treated with VL-CAR-T cells after 4 weeks of treatment, although there was no statistical difference compared to scFv-based CAR-T cells (**Figures 5D**, **S3B**). In addition, VL-CAR-T cells significantly prolonged the survival of mice compared to P1h2-CAR-T cells (**Figure 5E**).

To determine whether active CAR-T cells could be located at the tumor site, we constructed a T-cell tracking reporter by introducing a highly sensitive nano-luciferase element. The results showed that all CAR-T cells could be tracked and co-located with tumor cells *in vivo*. BLI of CAR-T cells suggested that the total flux of V_L_-based CAR-T cells was significantly increased after 17 days of CAR-T cell administration compared to other CAR-T treatment groups (**Figures 5F, G**).

### 3.6 VL-CAR-T cells eliminate tumors with greater safety *in vivo*

**Figure 6:**
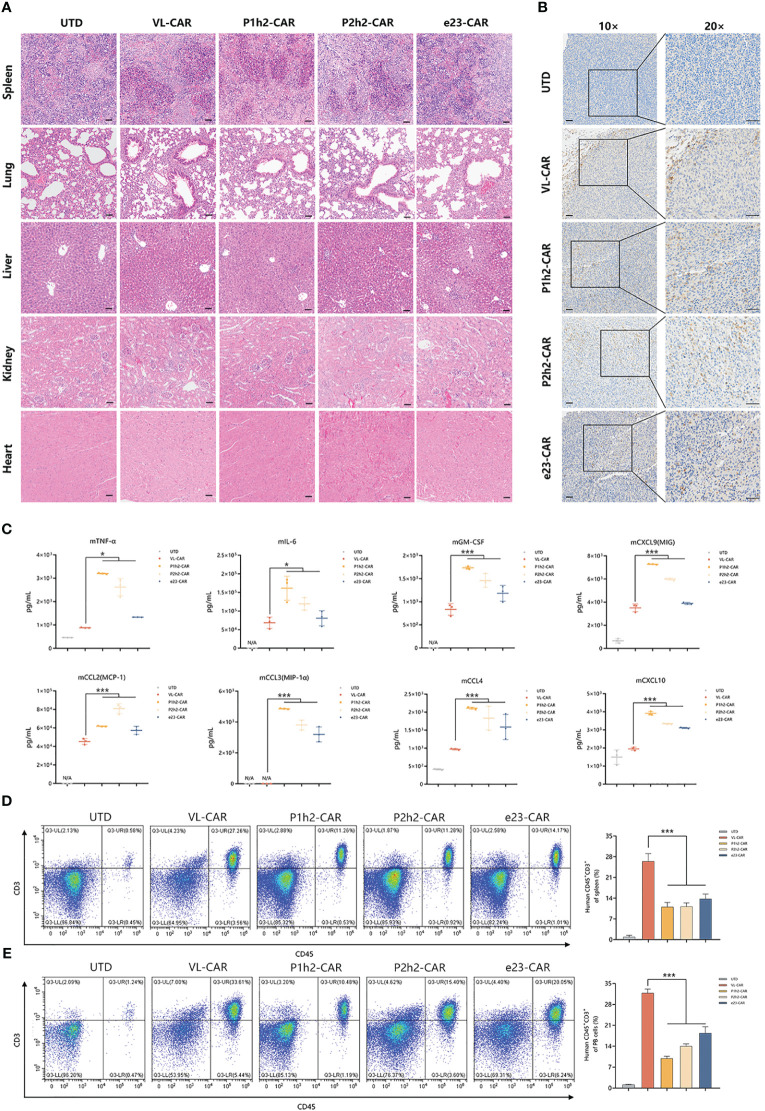
VL-CAR-T cells eliminate tumors with greater safety *in vivo*. **(A)** H&E staining of several main organs including spleen, lung, liver, kidney, and heart after 4 weeks of UTD/CAR-T cell injection. Scale bar=50μm. **(B)** Human CD3 expression in the tumor of mice after 17 days of UTD/CAR-T cell injection *in vivo*. Scale bar=50μm. **(C)** Analysis of murine cytokine secretion from mouse serum treated with UTD/CAR-T cells 17 days after injection. The number of T cells per microliter of spleen tissue **(D)** and milligram of peripheral blood (PB) **(E)** 35 days after injection. N/A, Not applicable. Asterisk indicates a statistically significant difference compared to the VL-based CAR-T group, *p ≤ 0.05, ***p ≤ 0.001.

To evaluate the safety of CAR-T cells, we measured the effects on several major organs and cytokines in mice treated with different CAR-T cells. According to the H&E results, there were no gross histological changes in the main organs examined in all CAR-T cell treatments (**Figure 6A**). Additionally, we observed distinct lymphoid structures in the spleen in all CAR-T cell treatment groups compared to the UTD group (**Figure 6A**).

In order to identify the specific location of CAR-T cells, we conducted immunohistochemistry staining. The results showed that human CD3^+^ cells efficiently infiltrated the tumor tissues in all CAR-T cell infection groups *in vivo* (**Figure 6B**). To further evaluate the safety of CAR-T therapy, we detected cytokines of both human and murine origins using multiplexed cytokine analysis. The cytokines from humans showed no signal, while several murine cytokines were measured after 17 days of CAR-T cell injection *in vivo* (**Figure 6C**, **Tables S1**, **S2**). The results showed that VL-CAR-T cells resulted in a significant decrease in the secretion of mTNFα, mIL-6, mGM-CSF, mCCL2, mCCL3, mCCL4, mCXCL9, and mCXCL10 compared with scFv-based CAR-T cells (**Figure 6C**). In addition, we observed that the percentage of CD45^+^CD3^+^ cells 35 days after CAR-T injection was significantly increased in the spleen and peripheral blood of mice in the VL-CAR-T treatment group compared with the other groups (**Figures 6D, E**, **S5**), suggesting VL-CAR-T cells displayed superior proliferation and persistence *in vivo*.

## 4 Discussion

Although CAR-T cells have revolutionized cancer immunotherapy, there is a critical need for the development of safer and more effective CAR-T cell approaches to achieve increased benefit. HER2, one of the most studied tumor-associated antigens (TAAs) is overexpressed in many tumors and acts as a potential target for cancer immunotherapy (6, 37). It is well accepted that compared with murine scFvs, the anti-tumor efficacy of CARs may be improved using a human binding moiety in tumor therapy (38). We previously demonstrated that humanized e23sFv improves HER2-targeting and tumor-killing capacities while reducing immunogenicity. Here, we extended this work by improving the design and function of humanized HER2-specific scFvs. We successfully constructed four groups of third-generation CAR-T cells, which differ only in their extracellular antigen-recognition domains, and achieved optimal immunotherapeutic properties using low-affinity CAR-T cells.

Clinical trials strongly imply that the response rate using CAR-T cells with relatively low affinity was higher than that in the high-affinity treatment groups (39). Affinity tuning via modification of CAR scFvs has emerged as an interesting approach to achieve an affinity sufficient for tumor recognition while sparing non-malignant cells with limited TAA expression (6, 21, 40). A previous study demonstrated that reducing the affinity of scFvs for HER2 exhibits equally robust anti-tumor activity with enhanced safety in CAR-T cells. In the present study, we first attempted to screen and construct four affinities of the antigen-recognition domain of CARs using different sdAbs or scFvs with K_D_ values of 26.5 nM, 4.51 nM, 1.71 nM, and 0.12 nM. The results showed that all CAR-T cells could precisely recognize and efficiently kill target-antigen-positive cells. Of note, sdAb-based VL-CAR-T cells exhibited better proliferative capacity and lower cytokine production than other higher-affinity CAR-T cells, which may decrease the risk of cytokine release syndrome (CRS) to some extent (41, 42). Hence, it is an applicable way that sdAbs with antigen-binding activity can be used to reduce the affinity of CAR-T cells, and they have the potential to maintain the anti-tumor capacity and decrease the risk of CRS.

Our *in vitro* data suggested that all affinities of CAR-T cells exhibit similar killing abilities after initial antigen exposure. Although the anti-tumor effects of low-affinity CAR-T cells may be slow, the low affinity of CAR-T cells exhibits long survival and maintenance of function in tumor lesions and produces durable effects, which are exhibited as a lower level of apoptosis, lower exhaustion, and a memory-like phenotype than the other scFv CAR-T cells after multiple challenges with antigen. The same results were observed *in vivo*, as low-affinity CAR-T cells exhibited superior proliferation and persistence in long-term treatment. Our findings are consistent with some recent studies that suggest that the results may be attributed in part to a “fly-kiss” mode of engagement (39, 43). Mechanistically, low-affinity CAR-T cells may shorten the duration of engagement with tumor cells due to a faster rate of dissociation. The transient break during the “off” time may rejuvenate and preserve the function of CAR-T cells (44). Hence, CAR-T cells can enjoy “a temporary break”, which allows them to kill tumor cells without being driven into exhaustion (39). Accordingly, low-affinity CAR-T cells in our present study possessed a long-lasting tumor elimination capability since these CAR-T cells may have a break time by disengaging from tumor cells to avoid exhaustion.

In addition, constant and intense engagement with antigens may result in the persistent activation of CAR-T cells, which is harmful in solid tumors (19, 45). The advantages of low-affinity CAR-T cells were also verified *in vivo* experiments. Although low-affinity CAR-T cells (VL-CAR-T cells) prolonged the tumor treatment process, the cells could precisely locate and retain reactivity to the growth of tumors that was at least as potent as the high-affinity CAR-T cells at the endpoint. However, we found that mice treated with high-affinity CAR-T cells died after treatment. One possible reason is that high-affinity CAR-T cells caused an increased release of cytokines *in vivo* due to their strong binding with antigens. It has been reported that elevated levels of inflammatory cytokines (such as IL-6, CXCL9, and CXCL10, which were all significantly increased in the serum of mice treated with high-affinity CAR-T cells in the current study), may increase the risk of developing cytokine storm and disorders (46, 47).

Our immunohistochemistry results show that all CAR-T cell treatment groups can efficiently infiltrate the tumor tissues. However, compared to high-affinity CAR-T cells, a long time was needed for low-affinity CAR-T cells to exert their function in tumor lesions in the current study. A similar experiment was conducted by Caraballo et al. (43), who found that low-avidity CAR-T cells were unable to eradicate oversized tumors. Hence, we hypothesize that low-affinity CAR-T cells with weak engagement may be insufficient to exert strong anti-tumor effects in large tumors. A possible way of combining with a checkpoint blockade was proposed as an interesting way to enhance the anti-tumor effects, considering that weak engagement provided by low-affinity CAR-T cells may enhance the effector function of T cells (43, 48).

Our results revealed a new role for CAR-T cell engineering to tune the scFv affinity of the CAR binding domain using the human sdAb sequence. The results also demonstrated that a moderate reduction of antigen binding affinity of CARs could maintain the anti-tumor efficacy and improve the safety of CAR both *in vitro* and *in vivo*. These proof-of-concept experiments lay the foundation for further development of sdAb-based CAR-T cells in clinical trials. However, a constant decrease in affinity beyond a threshold that may affect the reorganization and function of CAR-T cells in clinical trials may also be affected (39). Hence, there is a need for clinical trials to determine the therapeutic value of low-affinity anti-HER2 CAR-T cells. Moreover, tuning the affinity of scFvs is not the only way to improve the function of CAR-T cells. Combining other strategies such as the structure of the scFvs, the length of the hinge, the choice of the co-stimulatory domain, and the strength of the activation domain also need to be taken into account to enhance the performance of CAR-T cells in cancer immunotherapy (18, 49, 50).

## Data availability statement

The original contributions presented in the study are included in the article/**Supplementary Material**. Further inquiries can be directed to the corresponding authors.

## Ethics statement

All donors provided informed written consent and the use of human peripheral blood was approved (Approval ID: KY20214016-1) by the Ethics Committee of Fourth Military Medical University (Xi’an, China). The animal study was approved by Fourth Military Medical University Ethics Committee on Animal Care The study was conducted in accordance with the local legislation and institutional requirements.

## Author contributions

RZ: Data curation, Investigation, Project administration, Visualization, Writing – original draft. YC: Data curation, Investigation, Project administration, Writing – review & editing. YZ: Data curation, Project administration, Writing – review & editing. SL: Formal Analysis, Investigation, Writing – review & editing. XZ: Data curation, Formal Analysis, Writing – review & editing. YW: Investigation, Writing – review & editing. PW: Investigation, Writing – review & editing. RM: Investigation, Writing – review & editing. AY: Conceptualization, Funding acquisition, Writing – review & editing. BY: Conceptualization, Funding acquisition, Writing – review & editing, Supervision, Visualization.
