# Supplementary material for: Humanized single-domain antibody targeting HER2 enhances function of chimeric antigen receptor T cells
Source: Front Immunol. 2023 Nov 7;14:1258156. doi: 10.3389/fimmu.2023.1258156 (PMC10661930; doi:10.3389/fimmu.2023.1258156)
Supplement: Supplementary file 1 [file DataSheet_1.docx]

Supplementary Material

# Supplementary Figures and Tables

## Supplementary Figures

**
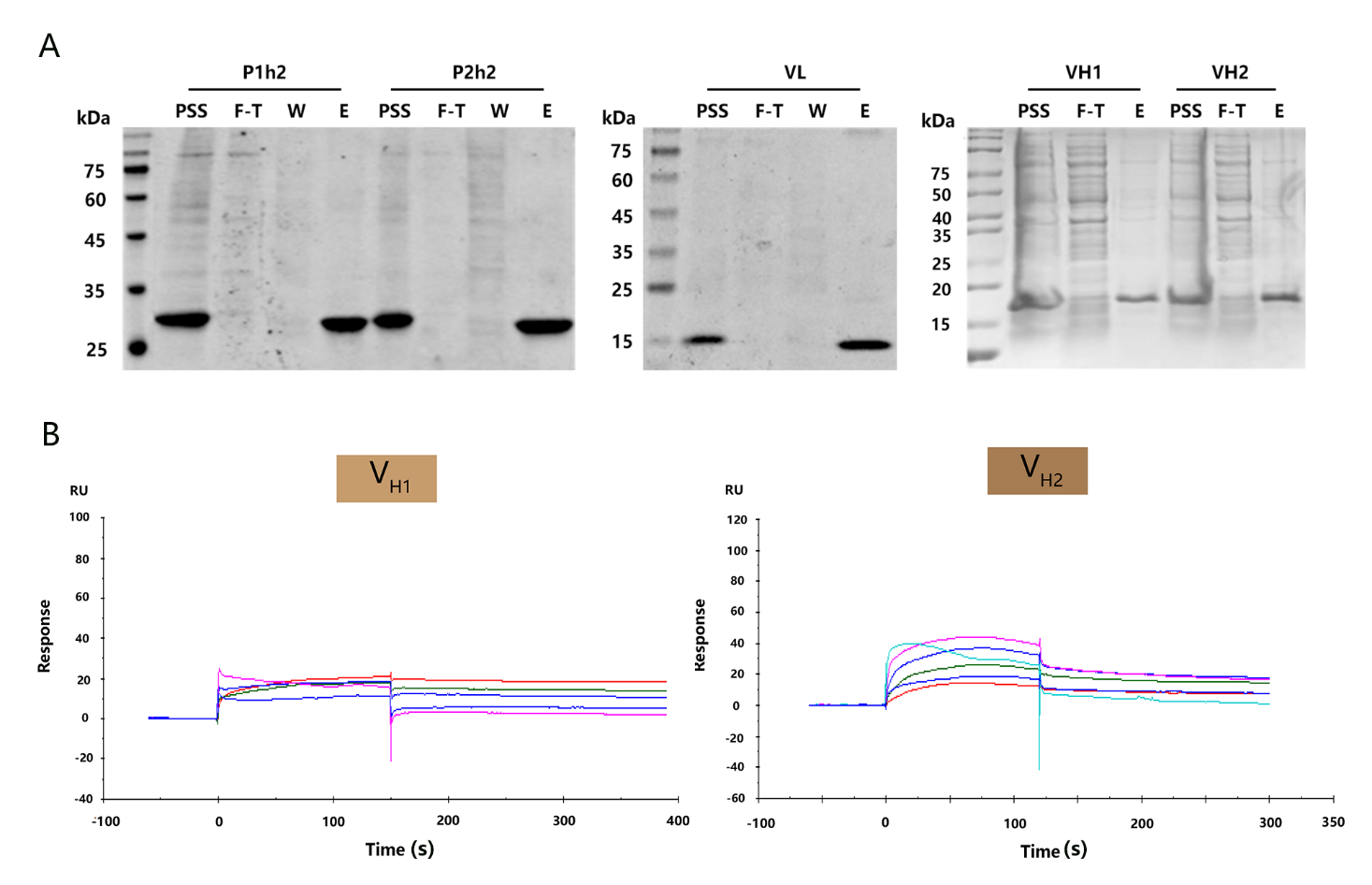
**

**Figure S1** (A) Coomassie brilliant blue G-250 dye. PSS: Protein stock solution, F-T: Flow-Through, W: Wash, E: Eluant. The expressions and kinetics of recombinant proteins in vitro. (B) SPR kinetic rates and apparent affinity binding constants of anti-HER2 scFvs in V_H1_, V_H2_ groups, respectively. All experiments were carried out at a constant flow rate of 10μL/min.


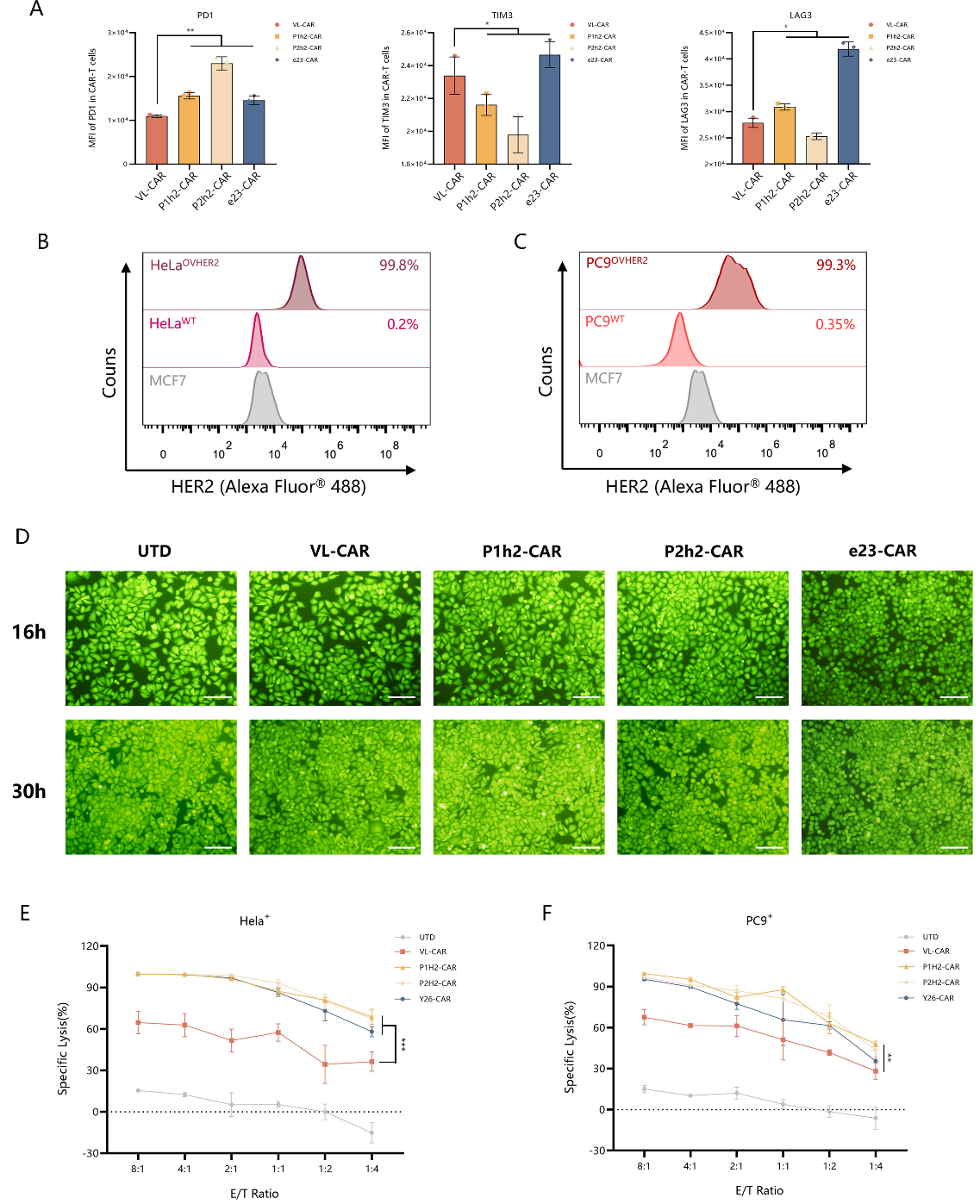


**Figure S2** (A) MFI of markers of inhibition in CAR-T cell using flow cytometry. HER2 expression in cell surface from wild type and overexpression HER2 in HeLa (B) and PC9 (C), respectively. (D) Representative images of CFSE in HER2^-^ tumor cells co-cultured with varies scFvs-based CAR-T at the E:T ratio of 2:1 for 16h and 30h. Scale bar=100 μm. The series of high- and low-affinity CAR-T cells were incubated with Firefly-Luciferase-transduced (E) HER2^+^ HeLa cells and (F) HER2^+^ PC9 cells at the indicated E:T ratio. Points represent mean cytotoxicity of replicate wells ± SD. Asterisk indicates a statistically significant difference compared to the V_L_-based CAR-T group, ^*^P ≤ 0.05, ^**^P ≤ 0.01, ^***^P ≤ 0.001.


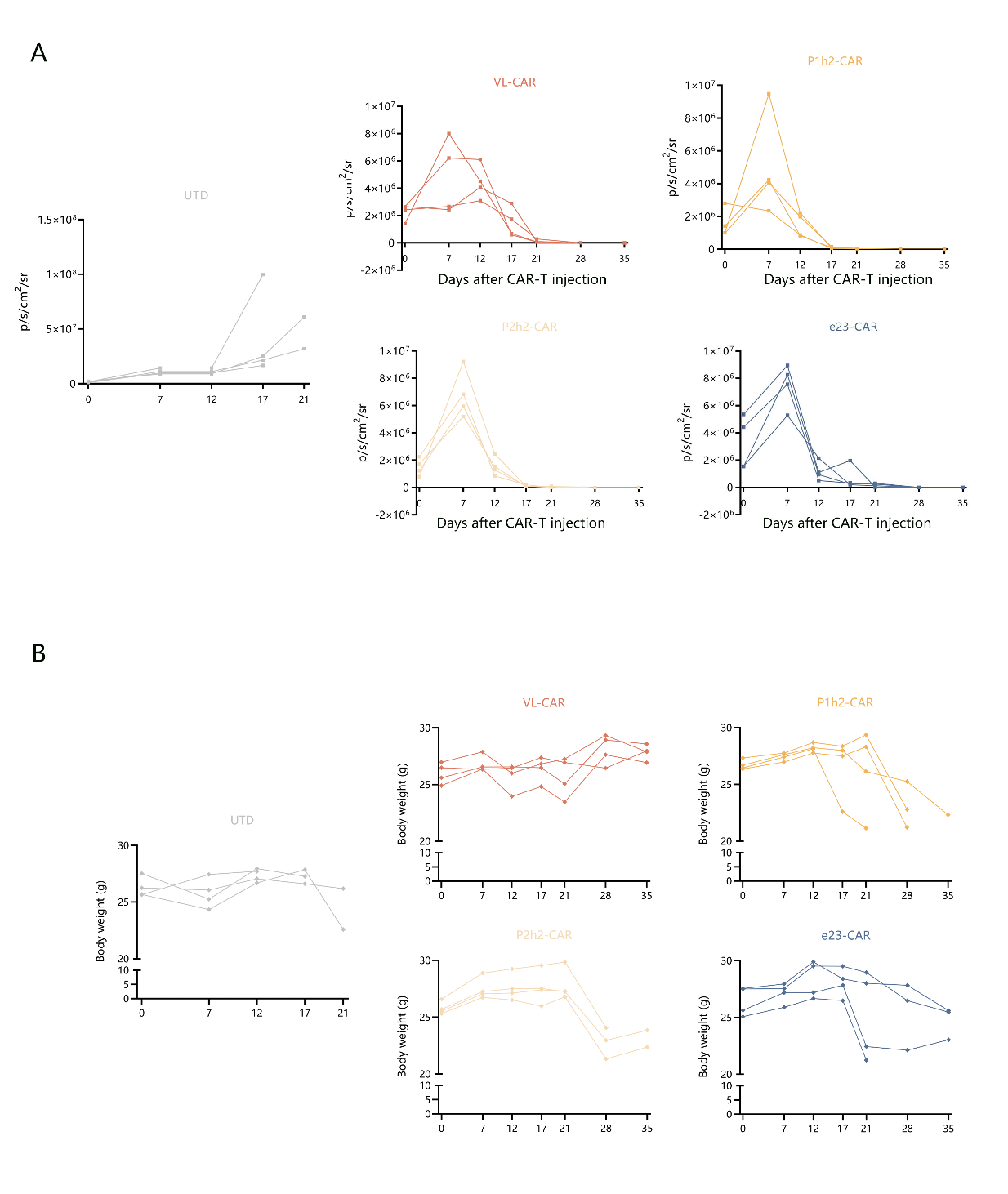


**Figure S3** CAR-T cells containing VL sdAb show better persistence and antitumor activity in vivo. (A) Tumor growth curves (average radiance) and (B) Body weight of each mouse treated with high- and low-affinity CAR-T cells and UTD. Each line represented one mouse in the corresponded group.


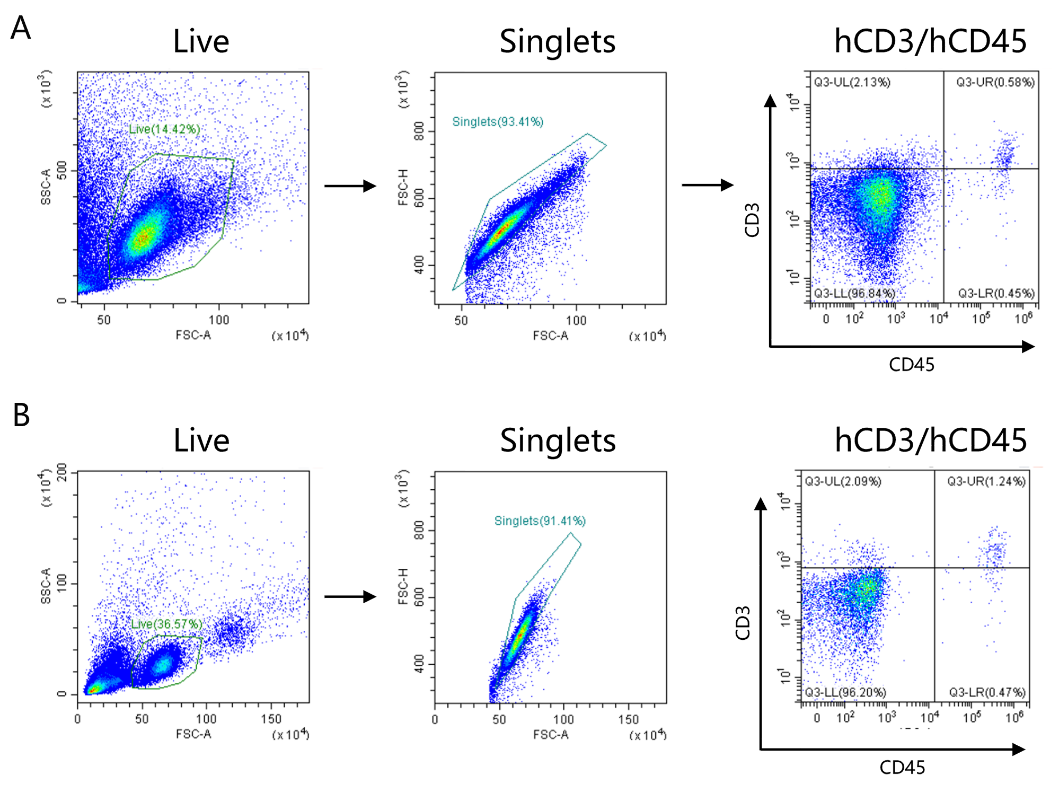


**Figure S4** Details on gating scheme utilized to analyze the percent of CD45+CD3+ cells in (A) spleen and (B) PB cells of mice in the day 35 after UTD/CAR-T cells injection.

## Supplementary Tables

|  | UTD | VL-CAR | P1h2-CAR | P2h2-CAR | e23-CAR |
| --- | --- | --- | --- | --- | --- |
| IL-4 | <11.12 | <11.12 | <11.12 | <11.12 | <11.12 |
| IL-2 | <3.819 | <3.819 | <3.819 | <3.819 | <3.819 |
| IP-10 | <0.97 | <0.97 | 2.17±1.27 | <0.97 | <0.97 |
| IL-1β | <10.05 | <10.05 | <10.05 | <10.05 | <10.05 |
| TNF-α | <2.23 | <2.23 | <2.23 | <2.23 | <2.23 |
| MCP-1 | <5.563 | <5.563 | <5.563 | <5.563 | <5.563 |
| IL-17A | <1.27 | <1.27 | <1.27 | <1.27 | <1.27 |
| IL-6 | <13.952 | <13.952 | 18.71±2.67 | <13.952 | <13.952 |
| IL-10 | <4.20 | <4.20 | <4.20 | <4.20 | <4.20 |
| IFN-γ | <4.582 | <4.582 | <4.582 | <4.582 | <4.582 |
| IL-12p70 | <7.12 | <7.12 | <7.12 | <7.12 | <7.12 |
| IL8 | <1.81 | <1.81 | 2.26±0.39 | <1.81 | <1.81 |
| TGF-β1 | 261.15±25.32 | 1320.84±20.93 | 1063.97±7.60^***^ | 1372.47±35.90^*^ | 1281.46±11.39 |

**Table. S1** Human cytokine release assay in vivo from mice serum after 17 days injection of different constructs of CAR-T cells

Data were expressed as mean values (±SD) (n=3). Asterisk indicates a statistically significant difference compared to the V_L_-based CAR-T group, ^*^*P* ≤ 0.05, ^***^*P* ≤ 0.001.

**Table. S2** Murine cytokine release assay in vivo from mice serum after 17 days injection of different constructs of CAR-T cells

|  | UTD | VL-CAR | P1h2-CAR | P2h2-CAR | e23-CAR |
| --- | --- | --- | --- | --- | --- |
| IFN-γ | <97.3 | <97.3 | <97.3 | <97.3 | <97.3 |
| IL-10 | <5364.4 | <5364.4 | <5364.4 | <5364.4 | <5364.4 |
| CCL4 | 461.93±3.64 | 882.63±12.79 | 3201.03±21.92^***^ | 2616.87±372.87^***^ | 1334.73±6.88^**^ |
| IFN-α | <231.5 | <231.5 | <231.5 | <231.5 | <231.5 |
| CXCL9 | 661.93±199.13 | 3510.60±334.75 | 7277.57±30.37^***^ | 5996.97±94.54^***^ | 3890.37±92.15^*^ |
| CXCL10 | 1499.33±402.17 | 1953.00±89.42 | 3914.00±96.38^***^ | 3342.00±25.36^***^ | 3109.33±36.46^***^ |
| TNF-α | <152.0 | 68445.07±15289.31 | 161158.82±32797.60^***^ | 119185.33±16854.75^*^ | 80439.33±20262.68^*^ |
| IL-6 | 661.93±199.13 | 3510.60±334.75 | 7277.57±30.37^***^ | 5996.97±94.54^***^ | 3890.37±92.15^*^ |
| VEGF | <113.5 | <113.5 | <113.5 | <113.5 | <113.5 |
| IL-4 | <89.3 | <89.3 | <89.3 | <89.3 | <89.3 |
| CCL3 | <224.6 | <224.6 | 4859.47±28.30^***^ | 3810.60±314.23^***^ | 3197.00±482.15^***^ |
| CCL2 | <445.5 | 45326.13±3216.10 | 61815.27±400.22^***^ | 80859.47±5318.40^***^ | 57362.73±4314.00^***^ |
| GM-CSF | <522.0 | 834.37±126.53 | 1735.13±31.23^***^ | 1459.37±150.61^***^ | 1182.70±170.94^**^ |

 Data were expressed as mean values (±SD) (n=3). Asterisk indicates a statistically significant difference compared to the V_L_-based CAR-T group, ^*^*P* ≤ 0.05, ^**^*P* ≤ 0.01, ^***^*P* ≤ 0.001.
